# Effects of human donor milk on gut barrier function and inflammation: *in vitro* study of the beneficial properties to the newborn

**DOI:** 10.3389/fimmu.2023.1282144

**Published:** 2023-11-08

**Authors:** Claudio Rodríguez-Camejo, Arturo Puyol, Paula Arbildi, Cecilia Sóñora, Laura Fazio, Gabriela Siré, Ana Hernández

**Affiliations:** ^1^ Área Inmunología, Departamento de Biociencias (DEPBIO), Facultad de Química, Universidad de la República, Montevideo, Uruguay; ^2^ Unidad Asociada de Inmunología, Instituto de Química Biológica (IQB), Facultad de Ciencias, Universidad de la República, Montevideo, Uruguay; ^3^ Laboratorio de Inmunología, Instituto de Higiene “Prof. Arnoldo Berta”, Universidad de la República, Montevideo, Uruguay; ^4^ Banco de Leche “Ruben Panizza”, Centro Hospitalario Pereira Rossell, Administración de los Servicios de Salud del Estado, Montevideo, Uruguay; ^5^ Escuela Universitaria de Tecnología Médica (EUTM), Hospital de Clínicas, Facultad de Medicina, Universidad de la República, Montevideo, Uruguay

**Keywords:** donor milk, human milk bank, gut inflammation, *in vitro* studies, Holder pasteurization

## Abstract

**Introduction:**

The gastrointestinal and immune systems of premature infants are not fully developed, rendering them more vulnerable to severe complications like necrotizing enterocolitis. Human milk offers a rich array of bioactive factors that collectively contribute to reducing the incidence of gut infections and inflammatory conditions. When a mother's milk is unavailable, preterm infants are often provided with donor human milk processed in Human Milk Banks. However, it remains uncertain whether pasteurized milk confers the same level of risk reduction as unprocessed milk. This uncertainty may stem from the well-documented adverse effects of heat treatment on milk composition. Yet, our understanding of the comprehensive impact on protective mechanisms is limited.

**Methods:**

In this study, we conducted a comparative analysis of the effects of raw versus pasteurized milk and colostrum versus mature milk on cellular functions associated with the gut epithelial barrier and responses to inflammatory stimuli. We utilized THP-1 and HT-29 cell lines, representing monocyte/macrophages and gut epithelial cells, respectively.

**Results:**

Our observations revealed that all milk types stimulated epithelial cell proliferation. However, only raw colostrum increased cell migration and interfered with the interaction between *E. coli* and epithelial cells. Furthermore, the response of epithelial and macrophage cells to lipopolysaccharide (LPS) was enhanced solely by raw colostrum, with a milder effect observed with mature milk. In contrast, both raw and pasteurized milk diminished the LPS induced response in monocytes. Lastly, we examined how milk affected the differentiation of monocytes into macrophages, finding that milk reduced the subsequent inflammatory response of macrophages to LPS.

**Discussion:**

Our study sheds light on the impact of human milk on certain mechanisms that potentially account for its protective effects against necrotizing enterocolitis, highlighting the detrimental influence of pasteurization on some of these mechanisms. Our findings emphasize the urgency of developing alternative pasteurization methods to better preserve milk properties. Moreover, identifying the key components critically affected by these protective mechanisms could enable their inclusion in donor milk or formula, thereby enhancing immunological benefits for vulnerable newborns.

## 1 Introduction

The superior nutritional value of Human Milk (HM) in newborns (NB) is widely acknowledged. The World Health Organization advocates exclusive breastfeeding for the initial six months of an infant’s life, followed by the gradual introduction of supplementary foods while continuing breastfeeding for at least two years (1). The provision of nutritional and bioactive elements in HM adjusts to the specific biological needs of NB. HM offers a well-suited and adaptable quantity of nutrients along with a multitude of bioactive constituents. Notably, a variety of these components contribute significantly to the defense against infections, development, and homeostasis of the immune system (2, 3). The aqueous fraction (AF) of HM contains the highest concentration and widest array of bioactive compounds. Within this fraction, prominent constituents such as lactoferrin, antibodies, α-lactalbumin, lysozyme, and Human Milk Oligosaccharides (HMO) exhibit the highest abundance (2–5). Furthermore, a diverse array of molecules are linked to milk fat globules, including lactadherin and mucins (6). Additionally, various components such as exosomes and microbiota are suspended in milk (7–9). Collectively, these components are believed to be responsible for the protective effects of human milk against infections and serious conditions associated with the underdeveloped gastrointestinal tract of premature infants (10–14). Necrotizing Enterocolitis (NEC) is one of the most devastating pathologies (15–17). The disruption of gut microbiota balance is believed to trigger this condition, initiating a key pathogenic mechanism involving the activation of Toll-like receptor 4 (TLR4) by lipopolysaccharides (LPS) (18). NEC has been correlated with certain attributes of the underdeveloped preterm gut, including: i) reduced production of mucus and antimicrobial molecules., ii) increased intestinal permeability associated with impaired epithelial regeneration, iii) elevated expression and activation of TLR4, and iv) compromised peristalsis (12, 19, 20). Numerous epidemiological studies have provided evidence for the protective impact of breastfeeding against NEC (17, 21, 22). However, only a limited number of studies have explored the potential mechanisms underlying the protective effects of human milk *in vitro*. Interestingly, it has been observed that colostrum can augment the response of epithelial cells to LPS, possibly due to its high concentration of sCD14 (23). Several *in vitro* and *in vivo* studies have examined the effects of the specific components present in human milk. For instance, IgA, lactoferrin, HMO, lactadherin, Trefoil Factor 3 (TFF3), exosomes, and lipids have been investigated for their potential effects on gut inflammation (24–30). Although these studies have provided valuable insights into the effects of specific components present in milk, it is important to note that human milk contains a complex mixture of hundreds of bioactive molecules present at varying concentrations. These molecules can interact synergistically, redundantly, or antagonistically, leading to intricate and multifaceted effects. As a result, the outcomes of these individual component studies may not fully capture the comprehensive impact of whole milk.

In situations where preterm NB are not able to receive their own mother’s fresh milk, an alternative approach for their feeding is donated HM through Human Milk Banks (HMB) (31). Nonetheless, epidemiological data indicate that the protective effect of donated HM against NEC does not reach the same level as that of fresh milk (21, 22, 32, 33). This disparity can be attributed to the potentially detrimental effects of milk processing within the HMB, as well as the distinctive properties of milk associated with different lactation stages owing to the dynamic nature of HM composition (2, 3). Colostrum has the highest concentration of proteins and a plethora of bioactive components (2–4, 34). However, most donations received by HMB consist of mature milk. To ensure microbiological safety, donor milk undergoes pasteurization using the Holder method, which involves heating at 62.5°C for 30 min (31). This treatment has a negative impact on milk composition, leading to variable reductions in the concentration of most bioactive molecules (4, 35). Despite this potential reduction in bioactive molecules due to pasteurization, donated HM still contains a considerable concentration of many bioactive components that play a role in providing passive immunization to newborns (4). Nevertheless, only a limited number of *in vitro* studies have been conducted to evaluate the protective effects of donated human milk (4, 36, 37). Currently, knowledge regarding the mechanisms and specific bioactive components responsible for the protective effects of donated human milk against intestinal inflammation is limited and incomplete.

The aim of this study was to enhance our understanding of the mechanisms underlying the beneficial effects of human milk in mitigating intestinal inflammation. Furthermore, we investigated how the Holder pasteurization method influences these properties using *in vitro* models. Our approach involved a comparative analysis of the effects of unprocessed and pasteurized colostrum and mature milk on the functionality of resident and recruited cells, which play pivotal roles in gut barrier integrity and inflammation control.

## 2 Materials and methods

### 2.1 Human donors and milk samples

Donated human milk (dHM) was collected from the Human Milk Bank (HMB) of Pereira Rossell´s Hospital in Montevideo (Uruguay) according to the Research Ethics Board of the Hospital guidelines. All donors had term delivery and met the HMB inclusion criteria (34); milk availability was an additional criterion for this study. Eleven individual samples of colostrum were obtained 3 days (median) after delivery (range:1–7), and 10 samples of mature milk were obtained at 6 months (median) of lactation (range:4–7). Aliquots of raw and Holder pasteurized milk (62.5°C for 30 min) were stored at −80°C until use. The aqueous fraction (AF) was obtained by two sequential centrifugation steps: 1,000 rpm for 10 min at 4°C to separate the cells and debris and 10,000 rpm for 30 min at 4°C to separate the fat (4). The endotoxin level was lower than 0.15 EU/mL in all AF samples, evaluated with the LAL Chromogenic Endotoxin Quantitation Kit (Thermo Fisher Scientific, Waltham, MA, USA). To minimize inter-individual variation, the AF obtained from the pooled samples was used for all experiments. The AF of these pooled samples was stored in aliquots at −80°C until use. For each *in vitro* assay, AF was diluted and sterilized by 0.2 μm filtration (GVS Filter Technology, Sanford, USA). IL-1β, IL-6, and TNF-α were not detected in the pooled samples analyzed by ELISA (Duoset ELISA kits, R&D Systems, Minneapolis, MN, USA).

### 2.2 Cell lines and reagents

The HT-29 and THP-1 cell lines (ATCC, Manassas VA, USA) were cultured at 37°C in a controlled atmosphere with 5% CO_2_ in complete medium: RPMI 1640 growth medium (Capricorn Scientific GmbH, Germany) supplemented with 10% heat-inactivated fetal bovine serum (FBS) (Capricorn Scientific), 100 µg/mL streptomycin and 100 U/mL penicillin (Sigma-Aldrich, St. Louis, USA). At 80%–90% confluence, HT-29 cultures were trypsinized, and the cells were counted and diluted according to each experimental design. Cultures of THP-1 at 1.0–1.2 × 10^6^ cells/mL were centrifuged, and the cells were counted and diluted according to each experimental design. Both cell lines were cultured for no more than 15 passages. Chemical and biological reagents were purchased from Sigma-Aldrich, unless otherwise specified.

### 2.3 *E. coli* and LPS-specific antibodies in aqueous human milk

The levels of bacteria and LPS-specific antibodies (IgA, IgG and IgM) were assessed by ELISA according to Zeng et al. (2018) with a few modifications (38). One colony of *E. coli* (ER2738; Lucigen Corp. Middleton, USA) were grown in 5 mL of Luria-Bertani (LB) broth (Mast Group Ltd., Liverpool, UK) overnight (ON) at 37°C with agitation (250 rpm) and centrifuged at 10,000 rpm for 15 min at 4°C. The pellet was washed twice with carbonate buffer (50 mM, pH 9.6) and heat inactivated at 65°C for 1 h. Then, OD_600 nm_ was adjusted to 0.5 and 100 μL/well was seeded in a high-binding 96-well ELISA plate (Thermo Fischer Scientific). For anti-LPS antibodies 100 μL/well of 2 μg/mL LPS (O26:B6) in carbonate buffer was seeded in the ELISA plate. After ON incubation at 4°C, the plates were washed with 0.05% Tween-20 in PBS and blocked with gelatin solution in PBS. Then, 100 μL/well of AF or commercial human colostrum IgA at 2-fold serial dilutions was incubated for 3 h at 37°C. The plates were washed and 100 μL/well of HRP-conjugated anti-human (IgA, IgG, or IgM) antibodies were incubated for 1 h at 37°C. Finally, the enzyme activity was developed as previously described (4) and the OD_450 nm_ was read (Labsystems Multiskan, Thermo Fisher Scientific).

### 2.4 Macrophage differentiation

THP-1 cells were differentiated into macrophage-like cells (dTHP-1) with phorbol 12-myristate 13-acetate (PMA), as described previously (39), with a few modifications. Briefly, cells were incubated with 50 ng/mL PMA in complete medium and after 3 days, the medium was removed, and the cells were rested for 24 h with fresh complete medium. To characterize the dTHP-1 macrophages obtained, the changes in morphology during PMA treatment were assessed by microscopy (Primovert Zeiss Microscopy GmbH, Germany), and FSC/SSC and mCD14 expression were assessed by flow cytometry (BD FACSCalibur™, New Jersey, USA). Cells were detached using Accutase (BioLegend, San Diego, CA, USA) and washed with PBS containing 0.1% BSA and 2 mM EDTA. The expression of surface CD14 was determined with a FITC-conjugated anti-human mCD14 antibody (BioLegend) using Propidium Iodide staining to exclude dead cells. Flowing 2.5.1. software (Turku Bioscience, Turku, Finland) was used for the data analysis. Detailed information about the cell density and volume of the growth medium for each experiment is described below.

### 2.5 Effect of LPS and milk aqueous fraction on cell viability

Viability was assessed using the (3-(4,5-dimethylthiazol-2-yl)-2,5-diphenyl-2H-tetrazolium bromide) MTT assay (40). For HT-29 cells, 100,000 cells in 200 μL of complete medium were seeded in 96-wells culture plates (Greiner Bio-One, Frickenhausen, Germany) and after 24 h the cells were washed with PBS. For dTHP-1, 100,000 THP-1 cells in 200 μL were differentiated in 96-well culture plates, as described above. Then, the cells in 200 μL of incomplete medium (without FBS) containing 1 μg/mL LPS were incubated for 24 h with or without 0.3% AF. Cells were washed with PBS, and 200 μL/well of 0.5 mg/mL MTT in PBS was incubated for 4 h, and the formazan crystals were solubilized with 150 μL/well of DMSO; the OD_560 nm_ was recorded. For THP-1 monocytes, 100,000 cells in 200 μL of incomplete medium with 1 μg/mL LPS in the presence or absence of 0.1% AF were seeded in U-bottom 96-well culture plates (Greiner Bio-One). After 24 h, the cells were centrifuged to remove the supernatant, and 200 μL/well MTT was incubated for 4 h. After centrifugation, the supernatant was carefully removed and the formazan crystals were solubilized with 170 μL/well of DMSO. A total of 150 μL/well was transferred to an F-bottom 96-well ELISA plate and the OD_560 nm_ was recorded. The results were normalized to the conditions in the absence of LPS and AF.

### 2.6 Effect of milk aqueous fraction on epithelial cell proliferation

Proliferation was assessed using the Crystal Violet (CV) method (41). Briefly, 5,000 HT-29 cells in 200 μL complete medium were incubated in 96-well culture plates (Greiner Bio-One) for 24 h. The cells were washed with PBS and AF (0.3%–3%) in incomplete medium, and 10% FBS was used as a positive control. After 24 h, the cells were fixed with 50 μL/well 5% formaldehyde in PBS for 15 min. The cells were then stained with 50 μL/well 0.05% CV solution for 10 min. The cells were washed several times with PBS and air-dried for 2 h. CV was solubilized with 150 μL/well of methanol for 20 min, and the OD_560 nm_ was recorded. The data were normalized to conditions in the absence of AF.

### 2.7 Effect of milk aqueous fraction on wound healing

Migration was assessed using the scratch assay, as previously described (42). Briefly, 550,000 HT-29 cells in 1 mL complete medium were seeded in 12-well culture plates (Greiner Bio-One). When the cells reached 90% confluence, injury was induced by scratching the monolayer with a sterile pipette tip. The cells were washed with PBS and incubated with AF (0.1% to 1.0%) in incomplete medium supplemented with 1% FBS. Wound healing was assessed using optical microscopy, and images were taken before and after 24 h of incubation (Primovert Zeiss Microscopy GmbH). In some experiments, mitomycin-C was used as a proliferation inhibitor. Wound healing (%) was calculated as 100 − [(A_24_/A_0_) × 100], where A_0_ and A_24_ are the wound areas before and after incubation, respectively. Fiji/ImageJ (open source software, Bethesda, USA) was used for the analysis of wound areas.

### 2.8 Effect of milk aqueous fraction on bacterial adhesion to epithelial cell

The effect of AF on *E. coli* and HT-29 interaction was evaluated according to Letourneau et al. (43), with a few modifications. Briefly, 250,000 HT-29 cells in 500 μL complete medium without antibiotics were incubated for 48 h in 24-well culture plates. The medium was discarded, and the cells were washed with PBS. One colony of *E. coli* (ER2738, Lucigen Corp.) was grown in 2 mL LB broth for 3 h–4 h at 37°C with agitation. The bacterial concentration was estimated by OD_600 nm_ (1 OD_600 nm_ ≅ 8 × 10^8^ bacteria/mL), and 500 μL of a bacteria; suspension in PBS was added to HT-29 (ratio 1:1) in the presence or absence of AF (1%–10%) or 100 μg/mL of commercial human colostrum IgA. After 3 h of incubation, the unattached bacteria were discarded and the cells were washed several times with PBS. Bacteria were detached with 100 μL/well of 1% Triton X-100 and 10-fold dilutions were incubated in LB agar plates at 37°C for 24 h to count the colonies. The data were normalized to the conditions in the absence of AF (maximum bacterial adhesion).

The effect of AF on bacterial proliferation has been investigated previously. Briefly, bacteria in PBS or LB broth with or without AF, were incubated for 4 h with agitation (250 rpm). The bacterial load was estimated at OD_600 nm_, as described above.

### 2.9 Effect of milk aqueous fraction on cell response to LPS

To evaluate the response of HT-29 cells to LPS, 200,000 cells in 500 μL complete medium were incubated for 48 h in a 24-well culture plate (Greiner Bio-One). For the dTHP-1 macrophages, 500,000 cells in 600 μL of complete medium were differentiated in a 24-well culture plate (Greiner Bio-One), as described in *Section 2.4*. Cells were washed with PBS, and 500 μL of incomplete medium containing 1 μg/mL LPS with or without AF (0.03%–0.3%) was added. After 24 h, the supernatant was collected and stored at −80°C until analysis. For THP-1 monocytes, 500,000 cells in 500 μL of incomplete medium containing 1 μg/mL LPS, with or without AF (0.01%–0.1%), were seeded in 24-well culture plates (Greiner Bio-One). After 24 h, the supernatant was obtained by centrifugation and stored at −80°C until analysis. The effects of sCD14 and human colostrum IgA (treated or not treated with Holder pasteurization) on LPS cell responses were assessed.

### 2.10 Cytokine determination

The concentrations of IL-1β, IL-6, IL-8, IL-10, TNF-α, and MIP-3α in the culture supernatants were measured in duplicate by ELISA using the corresponding Human Duoset ELISA kit (R&D Systems) according to the manufacturer’s instructions. Labsystems Multiskan MS (Thermo Fisher Scientific) plate reader was used to register OD_450 nm_. The lower concentration of the standard curve was 62.5 pg/mL for IL-1β, 9.4 pg/mL for IL-6, 31.2 pg/mL for IL-8, and IL-10, 15.6 pg/mL for TNF-α and MIP-3α.

### 2.11 Effect of milk aqueous fraction on macrophage differentiation

To study the impact of AF on macrophages (dTHP-1) differentiated from monocyte-like THP-1 cells, AF (0.03%–0.3%) was added during PMA treatment, and the differentiation process was studied as described above. The relative cell number after differentiation was assessed using the Crystal Violet method described in *Section 2.6*, using 20,000 THP-1 cells in 96-well culture plates. Viability was determined by MTT assay and the expression of mCD14 by flow cytometry, as described in Sections 2.5 and 2.4, respectively. The LPS (1 μg/mL) response of dTHP-1 was evaluated as described in Section 2.9.

### 2.12 Statistical analysis

In all studies, at least three analytical replicates were performed in three independent experiments. The results are expressed as the mean and Standard Error of the Mean (SEM) of independent experiments in the text, and results from one representative assay are shown in the figures. Pairwise comparisons performed made using Student’s t-test and one-way ANOVA with Tukey’s multiple comparisons *post-hoc* test for multiple comparisons. Paired comparisons of colostrum lactadherin content were performed using the Wilcoxon matched-pairs signed-rank test. GraphPad Prism (6.0 version) was used for statistical analysis. The statistical significance level was defined as 95% confidence and is indicated in the figures with asterisks: *p ≤0.05, **p ≤0.01, and ***p ≤0.001.

## 3 Results

### 3.1 Impact of donated milk on epithelial cell proliferation and migration

**Figure 1:**
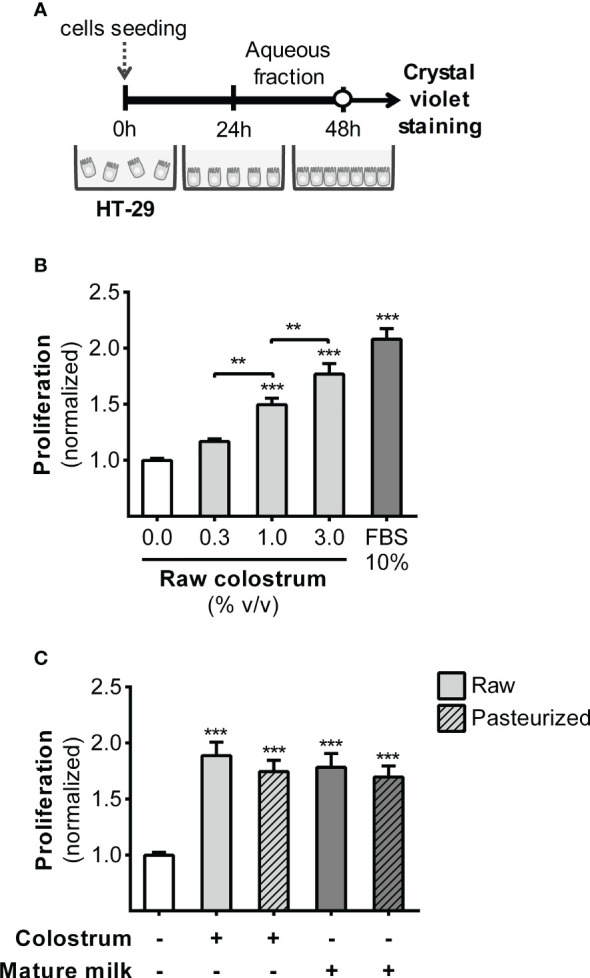
Effect of human milk AFs on gut epithelial cell proliferation. **(A)** Experimental design. **(B)** The effect of different concentrations of AF from raw colostrum on HT-29 cell proliferation. **(C)** Comparative effect of 3% AF derived from raw or pasteurized colostrum and mature milk on HT-29 cell proliferation. Data were normalized relative to the control condition (open bar). The bars represent the mean ± SEM of six analytical replicates, corresponding to one representative of three independent experiments. Asterisks on the bars indicate significant differences compared to the control condition, as determined by one-way ANOVA test (**p ≤0.01, ***p ≤0.001).

**Figure 2:**
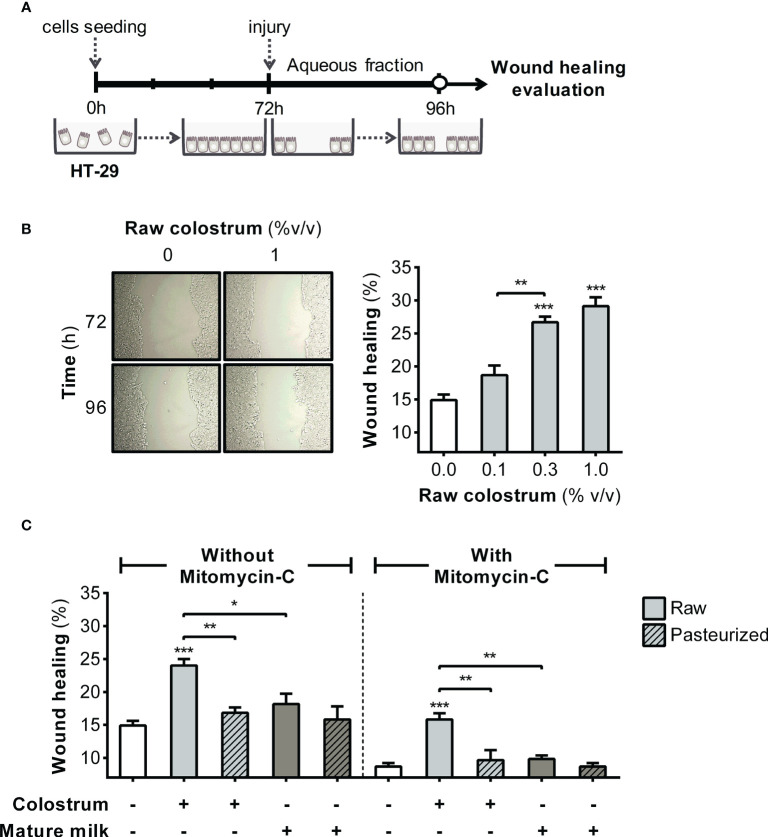
Effect of human milk AF on the wound healing of gut epithelial cells. **(A)** Experimental design. **(B)** Representative photograph (×10 magnification) illustrating the wound area before and after 24 h of incubation with raw colostrum. The graph depicts the dose-dependent effect of AF from raw colostrum on wound healing. **(C)** Comparative influence of 3% AF derived from raw or pasteurized colostrum and mature milk on wound healing, both in the absence and presence of 3 µg/mL mitomycin-C. Data were normalized relative to the control conditions (open bar). Bars represent the mean ± SEM of four analytical replicates, corresponding to one representative of three independent experiments. Asterisks on the bars denote significant differences in relation to the control condition, as determined by one-way ANOVA (*p ≤0.05, **p ≤0.01, ***p ≤0.001).

We first evaluated the effect of the AF of raw and pasteurized donated milk on strengthening of the epithelial barrier. This evaluation was performed by investigating the impact of milk AF on key cellular processes, such as proliferation and migration, using the HT-29 cell line. In this phase, our primary focus was to examine the effect of AF on cell proliferation (**Figure 1A**). As shown in **Figure 1B**, AF derived from raw colostrum exhibited a concentration-dependent effect on cell proliferation. Specifically, at a concentration of 3% AF, a significant fold-increase of 1.8 ± 0.2 (mean ± SEM) was observed compared to the basal condition. To ascertain whether this observed effect was unique to specific milk components, we conducted a comparative analysis by evaluating cell proliferation in the presence of Bovine Serum Albumin (BSA). BSA did not induce noticeable cell proliferation at any of the tested concentrations (**Figure S1A**). We compared the influence of the AF of raw and pasteurized colostrum and mature milk on cell proliferation. Interestingly, similar effects and magnitudes were observed for these distinct milk types (**Figure 1C**). A scratch assay was performed to assess the effect of milk on the migration of epithelial cells (**Figure 2A**). A concentration-dependent enhancement in wound healing was observed with the AF of raw colostrum (**Figure 2B**); a fold increase of 1.9 ± 0.1 (mean ± SEM) was noted with 1% v/v raw colostrum. To ascertain the specificity of this result for colostrum, we confirmed that BSA did not induce wound closure (**Figure S1B**). As wound healing relies on both cell migration and proliferation, we assessed the relative impact of raw and pasteurized colostrum and mature milk at a concentration of 0.3% AF, which did not stimulate cell proliferation (**Figure 1B**). Our observations revealed that only the AF of raw colostrum stimulated cell migration (**Figure 2C**), which was verified by employing mitomycin-C as an inhibitor of cell proliferation (**Figure 2C**).

### 3.2 Effect of donated milk on the interaction between bacteria and epithelial cells

**Figure 3:**
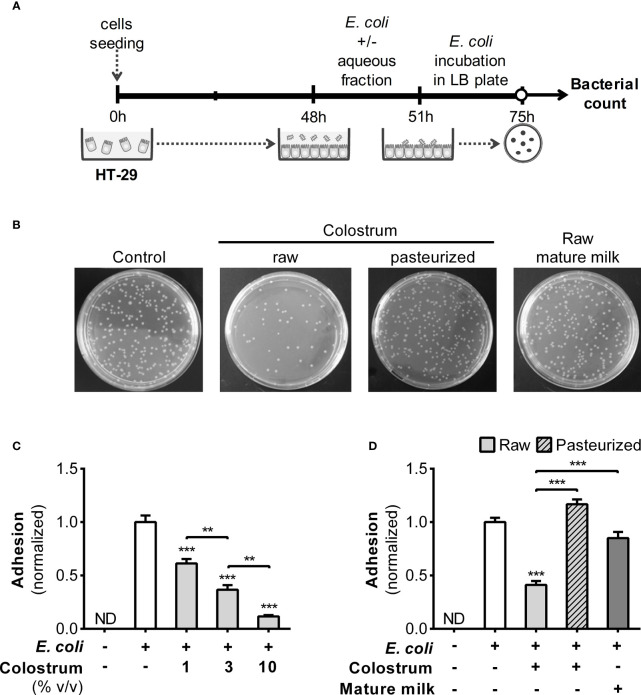
Effect of human milk AF on adhesion of *E. coli* to epithelial cells. **(A)** Experimental design. **(B)** Representative image of Luria–Bertani (LB) plates with or without the addition of 3% AF of colostrum or mature milk. **(C)** Dose-dependent influence of AF from raw colostrum on the interaction between *E. coli* and HT-29 cells (ratio 1:1). **(D)** Effect of 3% AF from raw and pasteurized colostrum as well as mature milk, on the interaction between *E. coli* and HT-29 cells. The data were standardized against the control conditions (open bar). Bars represent the mean ± SEM of four analytical replicates, corresponding to one representative of three independent experiments. The asterisks on the bars denote significant differences with respect to the control condition, as determined by one-way ANOVA (**p ≤0.01, ***p ≤0.001). ND, not detected.

Because the interaction between bacteria and the gut epithelium marks their initial entry into the lamina propria, inducing immune system activation, we assessed whether HM influences the adhesion of *E. coli* to HT-29 cells (**Figures 3A, B**). A concentration-dependent inhibition of bacterial adhesion was observed when colostrum AF was used (**Figure 3C**). The normalized adhesion ratio decreased from 1 (in the absence of AF) to 0.42 ± 0.03 (mean ± SEM) with 3% AF of raw colostrum. However, this inhibitory effect was not observed in the AF of pasteurized colostrum or mature milk (**Figure 3D**).

**Figure 4:**
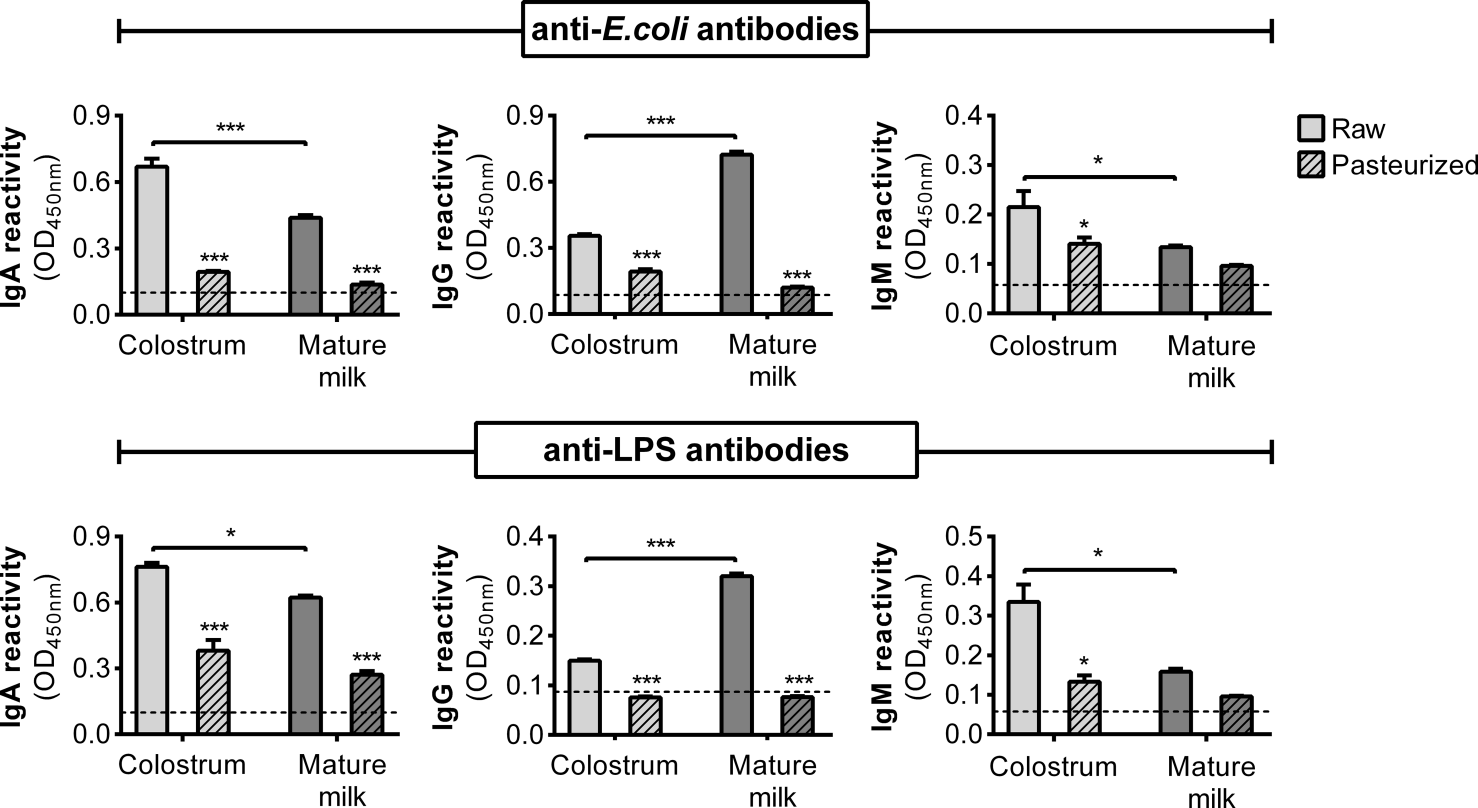
Specific antibodies against *E. coli* and LPS in the AF of human milk. Concentration of anti-*E. coli* and anti-LPS antibodies (IgA, IgG, and IgM) in raw and pasteurized colostrum and mature milk assessed by ELISA at non-saturating dilutions. The bars represent the mean ± SEM of the optical density at 450 nm based on data from four analytical replicates. Asterisks on the bars indicate significant differences between raw and pasteurized colostrum or mature milk, as determined by one-way ANOVA (*p ≤0.05, ***p ≤0.001).

Given the decreased bacterial adhesion, it is plausible that this effect may be due to the neutralizing influence of specific antibodies. Therefore, we quantified anti-*E. coli* and anti-LPS antibodies (IgA, IgG, and IgM) in HM using ELISA. Remarkably, colostrum exhibited elevated levels of specific IgA and IgM compared to those in mature milk, while IgG was predominantly detected in mature milk (**Figure 4**). The levels of antibodies across all isotypes were influenced by Holder pasteurization. These observations prompted us to investigate the effect of purified IgA from human colostrum on the adhesion of *E. coli* to epithelial cells. This commercial IgA displayed reactivity against *E. coli*, which was influenced by Holder pasteurization (**Figure S2A**). Consequently, when assessing their impact on bacterial adhesion, a reduction was observed in both untreated and treated IgA, although to a lesser extent following treatment (**Figure S2B**).

We controlled for the potential impact of AF on bacterial proliferation (**Figure S3A**); the presence of 30% AF did not exert any significant effect on bacterial growth (**Figure S3B**).

### 3.3 Effect of donated milk on the LPS-induced response of epithelial cells, macrophages, and monocytes

**Figure 5:**
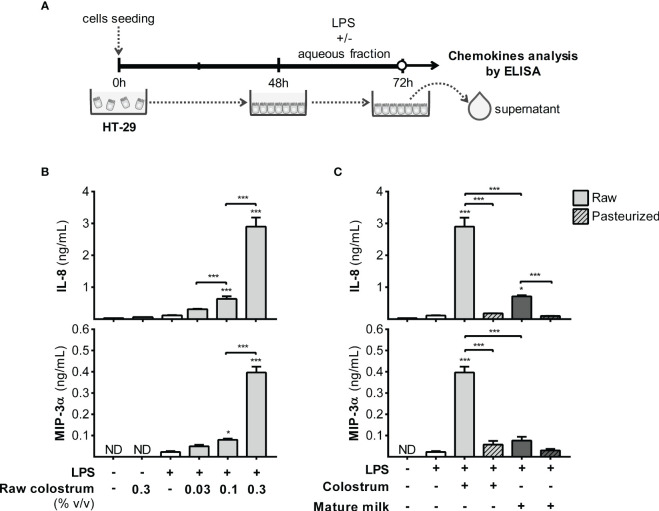
Effect of human milk AF on the LPS-response of gut epithelial cells. **(A)** Experimental design. **(B)** Dose-dependent effect of AF from raw colostrum on HT-29 chemokine response to 1 µg/mL LPS. **(C)** Comparative effect of 0.3% AF from raw or pasteurized colostrum and mature milk on HT-29 cells in response to 1 µg/mL LPS. The bars represent the mean ± SEM of three analytical replicates corresponding to one representative of three independent experiments. The asterisks on the bars indicate significant differences from the control condition (open bar, only LPS addition), as determined by one-way ANOVA (*p ≤0.05, ***p ≤0.001). ND, not detected.

LPS is among the most potent bacterial signaling molecules capable of eliciting an exaggerated inflammatory response in the gut of preterm newborns, involving resident and recruited leukocytes, as well as epithelial cells. We first focused on the response of HT-29 cells to LPS, both in the presence and absence of AF, over a 24-hour period. We assessed the production of IL-8 and MIP-3α chemokines in the supernatant (**Figure 5A**), which augmented the production of both chemokines in a concentration-dependent manner (**Figure 5B**). Stimulation with LPS in combination with 0.3% of AF from raw colostrum resulted in a substantial fold-increase of IL-8 and MIP-3α levels, specifically 12.9 ± 3.3 and 15.0 ± 1.4 (mean ± SEM) respectively, compared to the basal condition. However, when HT-29 cells were exposed to raw mature milk, the increase in IL-8 production was significantly lower (fold-increase of 5.1 ± 1.8, mean ± SEM) compared to colostrum incubation. Interestingly, this effect was abolished when colostrum or mature milk was subjected to Holder pasteurization (**Figure 5C**).

**Figure 6:**
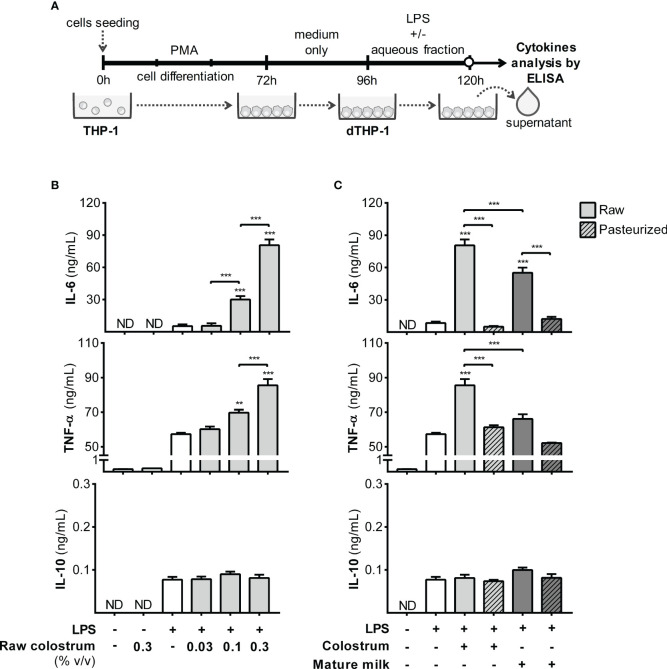
Effect of human milk AF on the LPS response of macrophages. **(A)** Experimental design. **(B)** Dose-dependent effect of AF from raw colostrum on dTHP-1 cytokine response to 1 µg/mL LPS. **(C)** Comparative effect of 0.3% AF from raw or pasteurized colostrum and mature milk on the dTHP-1 response to 1 µg/mL LPS. The bars represent the mean ± SEM of three analytical replicates, corresponding to one representative of three independent experiments. Asterisks on the bars indicate significant differences from the control condition (open bar, only LPS addition), as determined by one-way ANOVA (**p ≤0.01, ***p ≤0.001). ND, not detected.

Next, we examined the response of macrophages, a key resident cell type in the lamina propria responsible for orchestrating various aspects of the inflammatory process. To achieve this, we employed PMA-treated THP-1 cells (dTHP-1), a well-established *in vitro* model of macrophage-like behavior, to assess the influence of AF on cell response to LPS (**Figure 6A**). Initially, we confirmed that PMA incubation enhanced cell adhesion and increased the cell size, complexity, and expression of mCD14 (**Figure S4**). The addition of AF from raw colostrum increased the production of IL-6 and TNF-α in response to LPS. Specifically, a fold-increase of 9.0 ± 3.0 and 1.5 ± 0.1 (mean ± SEM) was observed for IL-6 and TNF-α levels respectively, when the cells were stimulated with LPS plus 0.3% AF. However, the IL-10 response to LPS remained unaltered (**Figure 6B**). The observed effect was significantly reduced when incubated with raw mature milk, resulting in a fold-increase of 4.9 ± 1.1 (mean ± SEM) for IL-6 levels. Notably, the effect observed with raw milk was abolished after Holder pasteurization (**Figure 6C**).

**Figure 7:**
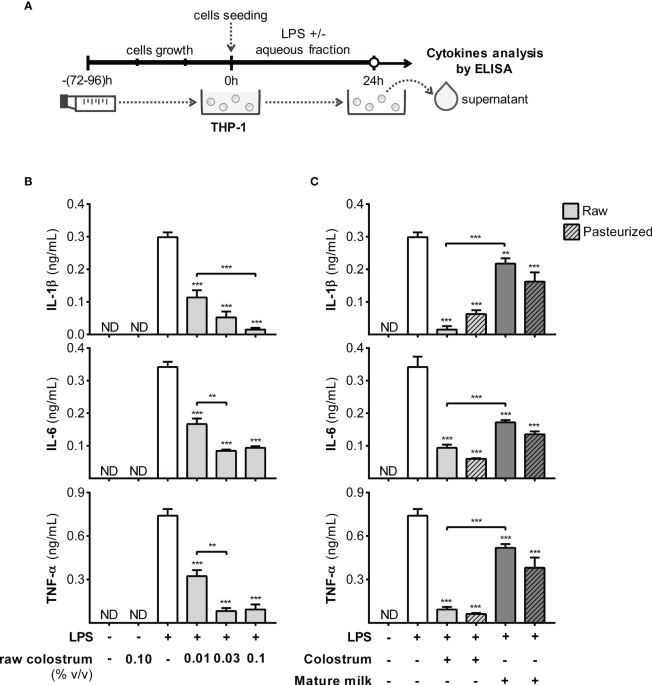
Effect of human milk AF on the LPS response of monocyte-like cells. **(A)** Experimental design. **(B)** Dose-dependent effect of AF from raw colostrum on the THP-1 cytokine response to 1 µg/mL LPS. **(C)** Effect of 0.1% AF from raw or pasteurized colostrum and mature milk on THP-1 response to 1 µg/mL LPS. The bars represent the mean ± SEM from three analytical replicates corresponding to one representative of three independent experiments. The asterisks on the bars indicate significant differences from the control condition (open bar, only LPS addition), as determined by one-way ANOVA (**p ≤0.01, ***p ≤0.001). ND, not detected.

Monocytes, recruited effector cells, play a crucial role in acute inflammation and have the potential to differentiate into macrophages. Therefore, we studied the effect of AF on the response of THP-1 monocyte-like cells to LPS (**Figure 7A**). Incubation with LPS in the presence of 0.1% AF of raw colostrum led to decreased levels of IL-1β, IL-6, and TNF-α, with fold decrease of 11.6 ± 4.8, 3.0 ± 0.4, and 6.2 ± 0.9, respectively (mean ± SEM) (**Figure 7B**). These effects were less pronounced with mature milk, resulting in fold decreases of 1.8 ± 0.3, 2.0 ± 0.3, and 2.5 ± 0.6 (mean ± SEM) for IL-1β, IL-6, and TNF-α, respectively. The effect of milk on the response of THP-1 monocytes to LPS was not altered by Holder pasteurization (**Figure 7C**).

We assessed cell viability following LPS incubation in our *in vitro* model and found that the viability of HT-29 cells remained unaffected. A minor reduction in the viability of dTHP-1 and THP-1 cells upon LPS stimulation was observed; however, this effect was not influenced by the addition of AF (**Figure S5**).

### 3.4 Effect of donated milk on the differentiation of monocyte to macrophage

**Figure 8:**
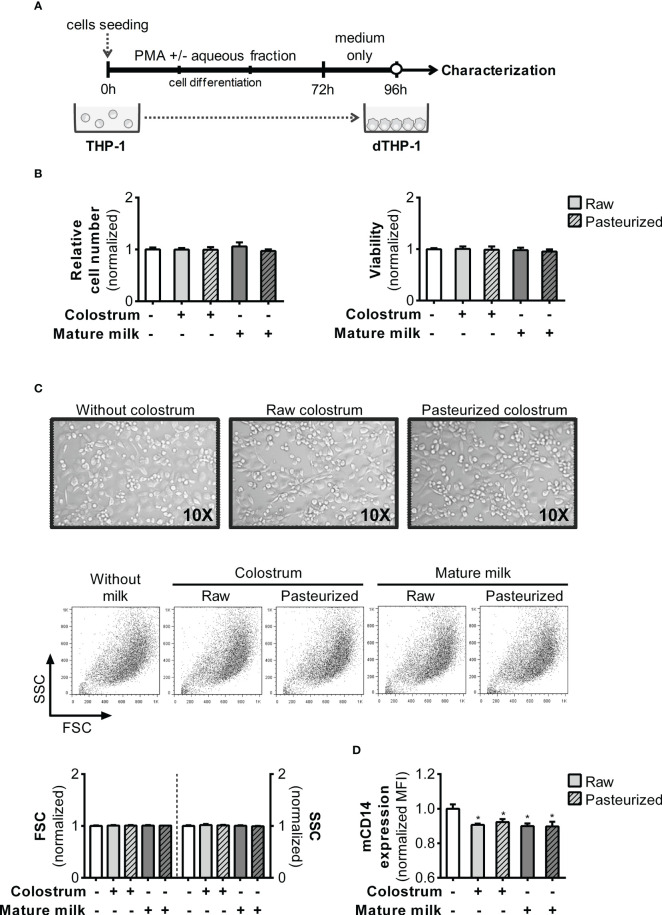
Effect of human milk AF on macrophage differentiation of PMA-treated monocyte-like cells. **(A)** Experimental design. **(B)** Effect of AF from raw or pasteurized colostrum and mature milk on the relative cell count and viability of macrophages (dTHP-1) on day 4. The effects were determined using the crystal violet assay and MTT method. **(C)** Representative photographs (×10 magnification) of cells and FSC/SSC Scatter on day 4 of culture, where cells were treated with PMA in the absence or presence of 0.3% AF from raw or pasteurized milk. **(D)** Flow cytometric analysis of mCD14 expression in dTHP-1 cells differentiated in the absence or presence of 0.3% AF from raw or pasteurized milk. The bars represent the mean ± SEM of four analytical replicates corresponding to one representative of three independent experiments. Asterisks on the bars indicate significant differences from the control condition (open bar), as determined by one-way ANOVA (*p ≤0.05).

The functional phenotype of monocyte-derived macrophages depends on their microenvironments. Therefore, we examined whether inclusion of 0.3% AF during PMA treatment could alter the attributes of the resulting dTHP-1 cells (**Figure 8A**). Initially, we ensured that the cell count and viability remained unaffected by PMA treatment and AF under all experimental conditions after 24 h of resting (**Figure 8B**). Moreover, based on the cell FSC/SSC parameters, the morphology of dTHP-1 cells was similar across all conditions (**Figure 8C**). The expression of mCD14, a differentiation marker, was slightly decreased in dTHP-1 cells cultured with AF from all milk types compared to the basal condition (**Figure 8D**).

**Figure 9:**
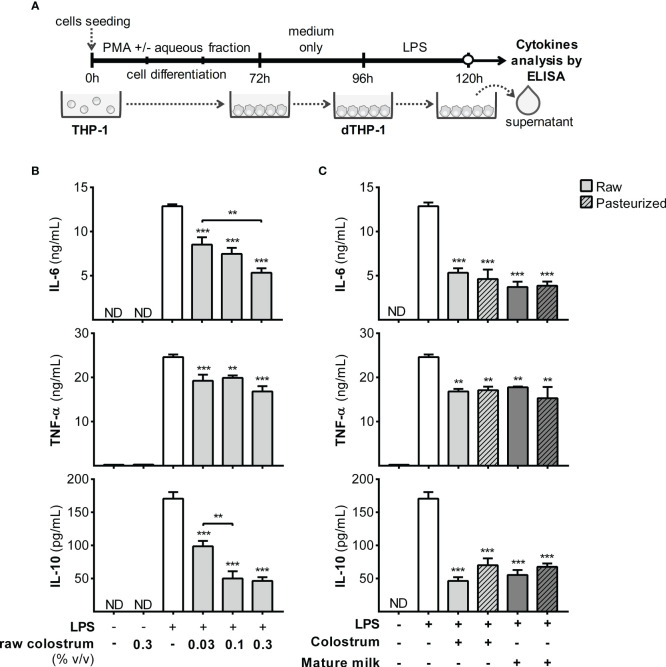
Effect of human milk AF on the LPS response of dTHP-1 during PMA-driven macrophage differentiation. **(A)** Experimental design. **(B)** Dose-dependent effects of AF from raw colostrum during macrophage differentiation on the cytokine response of dTHP-1 cells to 1 µg/mL LPS. **(C)** Comparative effect of 0.3% AF from raw and pasteurized milk during macrophage differentiation on the cytokine response of dTHP-1 cells to 1 µg/mL LPS. The bars represent the mean ± SEM of three analytical replicates corresponding to one representative of three independent experiments. Asterisks on the bars indicate significant differences from the control condition (open bar, differentiation without AF), as determined by one-way ANOVA (**p ≤0.01, ***p ≤0.001). ND, not detected.

Next, we assessed the response of dTHP-1 cells obtained under various conditions (**Figure 9A**). dTHP-1 cells differentiated in the presence of 0.3% AF from raw colostrum displayed a dose-dependent reduction in cytokine production compared to the control condition. Specifically, the fold decreases were 2.8 ± 0.4 for IL-6, 3.7 ± 0.3 for IL-10, and 1.6 ± 0.1 for TNF-α (mean ± SEM) (**Figure 9B**). The addition of 0.3% AF from mature milk resulted in a reduced response to LPS, similar to that observed in colostrum. Moreover, the effect of AF from raw colostrum and mature milk on the LPS response remained unaffected by pasteurization (**Figure 9C**).

## 4 Discussion

**Figure 10:**
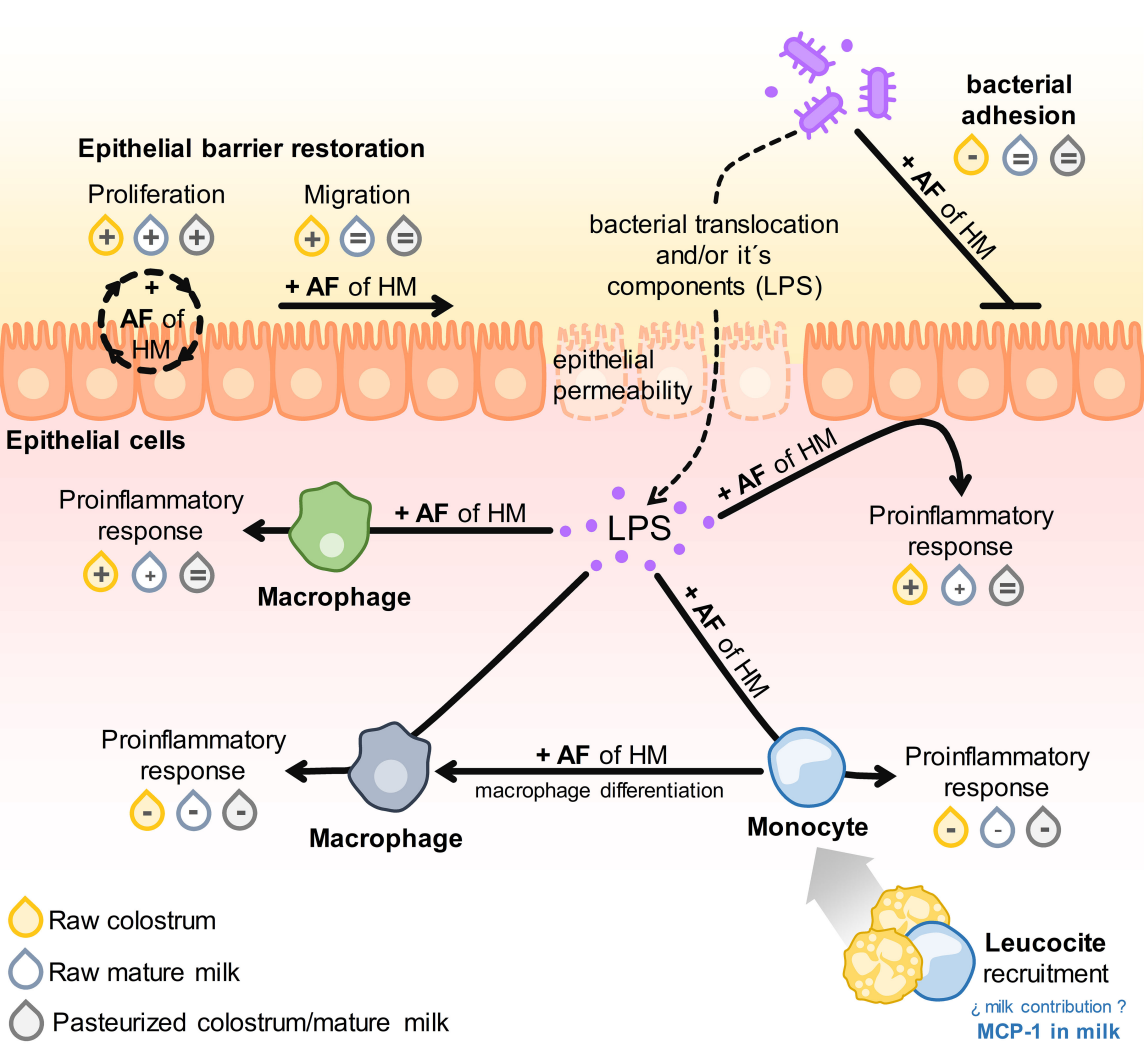
Schematic illustration of the results obtained from all assays. The impact of adding AF from raw or pasteurized colostrum and mature milk on the respective parameters for each assay is denoted as: enhancement (+), inhibition (−), or no difference (=) compared to the conditions without AF.

Numerous *in vivo* and *in vitro* experimental studies have demonstrated the beneficial effects of certain bioactive components in human milk (26, 27, 30, 44, 45). Nonetheless, limited research has delved into the mechanisms involved in the protective effects of donated HM within the neonatal gastrointestinal tract (4, 36, 37). The present study aimed to further investigate the biological significance of compositional changes in the activities of gut epithelial and innate immune cells upon exposure to bacterial stimuli. To mitigate the influence of inter-individual variability in milk composition on outcomes, we employed pooled samples of colostrum or mature milk collected the six-month after parturition, both in untreated and pasteurized conditions, as representative specimens of these lactation stages. Overall, our findings demonstrate that colostrum exerts more pronounced effects in most assessments than mature milk, consistent with its higher concentration of extensively documented bioactive components (2–4). Conversely, diverse effects of pasteurization were noted depending on the specific cell type and function under examination. These variations might be attributed to discrepancies in the interplay between the receptor profiles of distinct cell types and AF composition. The results are summarized in **Figure 10**.

Given the association between inadequate epithelial restoration and an immature gut, we initially investigated the impact of AF on the rates of proliferation and migration of HT-29 cells. Remarkably, AF from all milk sources triggered equivalent enhancement in cell proliferation. One potential bioactive constituent accounting for this effect could be Epidermal Growth Factor (EGF) (46), which maintains a consistent concentration throughout the first year of lactation and demonstrates resilience to the influence of Holder pasteurization (4, 35). AF from raw colostrum exhibited the capacity to stimulate cell migration. Within the wealth of bioactive constituents present in AF, lactadherin (MFG-E8) is a pivotal mediator of the wound healing process (25, 47–49). Consistent with our findings, lactadherin was more abundant during the early stages of lactation (50), and its levels were significantly affected by the Holder pasteurization process (**Figure S6**).

The attachment of bacteria and their subsequent invasion into epithelial cells plays a pivotal role in gut inflammatory pathologies. Our study revealed that only the AF of raw colostrum affected the interaction between *E. coli* and HT-29 cells. This effect can be attributed to the presence of various heat-sensitive bioactive components, including antibodies. Notably, we found that all anti-*E. coli* and anti-LPS antibodies decreased after pasteurization. Given that secretory IgA (sIgA) is the most abundant antibody isotype in human milk, we investigated the effects of purified colostrum sIgA on bacterial adhesion. Thermal treatment reduced the neutralizing effect of sIgA on bacterial adhesion to epithelial cells (**Figure S2**). These variations in the effects observed between the pasteurized and raw samples can be attributed to these underlying factors. Lactoferrin, a significant protein in AF, could also play a role in these outcomes (51), considering its sensitivity to thermal treatment and its decline over the course of lactation (52, 53).

The pathophysiology of NEC is closely linked to increased reactivity of the preterm gut epithelium to LPS. This reactivity is characterized by elevated TLR4 expression, translocation of LPS to the lamina propria, and the subsequent activation of inflammatory cells (18, 20). Globally, milk exhibits anti-inflammatory properties, while also playing a role in the effector mechanisms involved in defense against pathogens (54, 55). The ultimate outcome *in vivo* depends on the functional characteristics of resident cells in the lamina propria, signaling within the microenvironment, and the maturity of both the immune system and gut epithelium. In our *in vitro* model of gut epithelial cells and macrophages, we successfully replicated the role of raw milk in augmenting the inflammatory response to bacterial stimuli. In a study by LeBouder et al., it was observed that the response of HT-29 cells to LPS was reversed by employing a blocking antibody against sCD14 (23). CD14 exists in both the cell membrane-bound form (mCD14) and soluble form (sCD14), both of which can modulate the TLR4 signaling pathway (56, 57). In our previous study, we demonstrated that sCD14 is present in higher concentrations in colostrum than in mature milk, and its levels are drastically diminished by the Holder pasteurization process (4). We investigated the influence of thermal treatment on the functionality of recombinant sCD14 and found that the augmented LPS response in HT-29 and dTHP-1 cells was abolished by Holder pasteurization (**Figure S7**).

The severity of NEC is correlated with a decrease in circulating monocyte levels because of their recruitment into the gut (58); this parameter was proposed as a biomarker of NEC pathology (59). Consequently, we assessed the effect of AF on the response of THP-1 monocyte-like cells to LPS. Unlike epithelial and macrophage cells, this response was dampened by AF and remained unaffected by thermal treatment. However, the effect of purified sCD14 mirrored that observed in the other cell types (**Figure S7**). This distinct pattern indicated the involvement of other milk constituents in the LPS response. To investigate this further, we examined the effect of purified colostrum sIgA with reactivity to LPS on the response to this stimulus. No variation was observed across any of the cell types (data not shown). Lactoferrin is a crucial thermosensitive component that interacts with LPS and may contribute to modulating the response. In addition to the components that can directly interact with LPS, numerous bioactive molecules present in HM can either enhance or inhibit TLR expression and its associated signaling pathways (60). Ultimately, the distinct intrinsic properties of cell types, such as activation thresholds, patterns of receptor expression, and functional attributes, could elucidate the variations observed in the response to LPS.

Macrophages exhibit remarkable plasticity, and their responses can pivot between inflammatory and anti-inflammatory states based on cues from the microenvironment, such as M1- and M2-like phenotypes, in a simplistic view (61). We investigated whether AF might have an impact on macrophage behavior, potentially altering their responsiveness to LPS-induced inflammation. Interestingly, when AF was present during PMA-induced differentiation, a subdued inflammatory reaction to LPS was observed. While this outcome deserves more in-depth exploration of the relationship between macrophages and a potential M2-like phenotype, our findings are in line with prior research that showed the potential of pasteurized AF to redirect murine macrophages to an M2 phenotype (62). The milk constituents responsible for this effect remain unclear. Our findings suggest the involvement of bioactive components that remain unaffected by thermal processing, as evidenced by the similar outcomes in both raw and pasteurized samples.

Among these components, HMOs comprise a complex array of multifunctional elements that can potentially influence the outcomes of our *in vitro* experiments (63). The overall quantity of HMO is most abundant in colostrum, although the contribution of specific molecules within this group tends to vary throughout the lactation period (5). HMO are not affected by thermal treatment (64), which could explain some similar effects between the AF of raw and pasteurized milk. In addition, exosomes are another class of bioactive components present in the AF of milk that have shown *in vitro* and *in vivo* effects (30, 65, 66). However, their concentration decreases as lactation progresses, and they are influenced by Holder pasteurization (67). These and other components might potentially contribute to some of the effects observed in the AF; however, additional research is necessary to unravel their specific roles.

### 4.1 Limitations of this study

This study had several limitations from the perspective of knowledge translation. First, it employs simplistic *in vitro* models using transformed cell lines, which lack the complexity of tissue-microenvironment signals. Incorporating microbiota and mucus could provide a more accurate representation of the intricate gastrointestinal tract environment. Second, the observed *in vitro* effects may differ when the impact of gastrointestinal digestion on bioactive components is considered. Third, the study focused solely on the bioactive constituents of AF, excluding the potential effects of other milk components.

In conclusion, this study sheds light on the principal biological processes influenced by milk, which exhibit varying responses depending on lactation stage and thermal treatment. Further studies are underway to assess the relevance of our results in more complex *in vitro* and *in vivo* systems. This basic knowledge is essential for understanding the mechanisms underlying diverse clinical outcomes in preterm NB who are nourished with donor milk. Moreover, it provides insights into enhancing the strategies of Human Milk Banks in cases where fresh maternal milk is not accessible.

## Data availability statement

The original contributions presented in the study are included in the article/**Supplementary Material**. Further inquiries can be directed to the corresponding author.

## Ethics statement

The studies involving humans were approved by Comité de Ética en Investigación, Centro Hospitalario Pereira Rossell, Montevideo, Uruguay. The studies were conducted in accordance with the local legislation and institutional requirements. The participants provided their written informed consent to participate in this study.

## Author contributions

CR-C: Conceptualization, Formal Analysis, Funding acquisition, Investigation, Methodology, Project administration, Resources, Validation, Visualization, Writing – original draft, Writing – review & editing, Data curation. AP: Investigation, Methodology, Resources, Validation, Writing – review & editing. PA: Investigation, Methodology, Resources, Validation, Writing – review & editing. CS: Investigation, Methodology, Resources, Validation, Writing – review & editing. LF: Resources, Writing – review & editing. GS: Resources, Writing – review & editing. AH: Conceptualization, Formal analysis, Funding acquisition, Investigation, Methodology, Project administration, Resources, Supervision, Validation, Visualization, Writing – original draft, Writing – review & editing.
