# Supplementary material for: Effects of human donor milk on gut barrier function and inflammation: in vitro study of the beneficial properties to the newborn
Source: Front Immunol. 2023 Nov 8;14:1282144. doi: 10.3389/fimmu.2023.1282144 (PMC10663376; doi:10.3389/fimmu.2023.1282144)
Supplement: Supplementary file 1 [file DataSheet_1.docx]

Supplementary Material

Effects of human donor milk on gut barrier function and inflammation: in vitro study of the beneficial properties to the newborn.

**Claudio Rodríguez-Camejo^1,2,3^, Arturo Puyol^4^, Paula Arbildi^1,2,3^, Cecilia Sóñora^1,2,3,5^, Laura Fazio^4^, Gabriela Siré^4^, Ana Hernández^1,2,3,*^.**

1. Área Inmunología, Departamento de Biociencias (DEPBIO), Facultad de Química, Universidad de la República. Montevideo, Uruguay.

2. Unidad Asociada de Inmunología, Instituto de Química Biológica (IQB), Facultad de Ciencias, Universidad de la República. Montevideo, Uruguay.

3. Laboratorio de Inmunología, Instituto de Higiene “Prof. Arnoldo Berta”, Universidad de la República. Montevideo, Uruguay.

4. Banco de Leche Humana “Dr. Ruben Panizza”, Centro Hospitalario Pereira Rossell, Administración de los Servicios de Salud del Estado, Montevideo 11600, Uruguay.

5. Escuela Universitaria de Tecnología Médica (EUTM), Hospital de Clínicas, Facultad de Medicina, Universidad de la República. Montevideo, Uruguay

***Correspondence**:

Ana Hernández (aherna@fq.edu.uy)





**Figure S1. Effect of protein content on proliferation and wound healing of epithelial cells.** A solution containing BSA at a concentration of 20 mg/mL was utilized as a non-specific protein at the same concentration range as AF from raw colostrum in the assays. (A) The effect of BSA on HT-29 proliferation was evaluated, using 3% AF of raw colostrum as a positive control. Data are presented as normalized to the control condition (open bar). (B) The impact of BSA on HT-29 wound healing was examined, with 1% AF of raw colostrum employed as a positive control. The results are depicted as the mean ± SEM of six (proliferation) or four (wound healing) analytical replicates. Asterisks on the bars indicate significant differences compared to the control condition (open bar), determined by one-way ANOVA test (***p ≤ 0.001).





**Figure S2. Effect of sIgA on *E. coli* and epithelial cells interaction.** (A) The influence of thermal treatment (62.5°C, 30 min) on the *E. coli*-specific reactivity of sIgA purified from human colostrum at various concentrations is demonstrated. The data are presented as the mean ± SEM of optical density at 450nm for four analytical replicates. The asterisks on each point indicate significant differences in the reactivity of thermally treated versus non-treated sIgA, determined by the Student t-test (***p ≤ 0.001). (B) The impact of thermal treatment on the inhibitory effect of sIgA against *E. coli* adhesion to HT-29 cells is illustrated. The data are normalized against the control condition (white bar). Bars represent the mean ± SEM from four analytical replicates corresponding to one representative of two independent experiments. The asterisks on the bars indicate significant differences compared to the control condition, determined by one-way ANOVA test (**p ≤ 0.01, ***p ≤ 0.001).





**Figure S3. Effect of the AF of human milk on the *E.coli* proliferation.** (A) Experimental design. (B) Illustration of the impact of 30% AF from raw or pasteurized colostrum and mature milk on the proliferation of E. coli in both PBS and LB broth during 4 hours of incubation at 37°C. The results are expressed as the mean ± SEM from four separate analytical replicates. Asterisks on the bars denote significant differences compared to the initial bacterial load (open bar), determined by a one-way ANOVA test (***p ≤ 0.001).





**Figure S4. Monocyte derived macrophage differentiation.** (A) Overview of Experimental Design. (B) Depiction of monocyte-like THP-1 and macrophage-like dTHP-1 cells through representative images. The scatter plots of Forward Scatter (FSC) versus Side Scatter (SSC) display the alterations in morphology during the differentiation process. The data from flow cytometry analysis, encompassing parameters of morphology and surface CD14 expression (mCD14), are presented as the mean ± SEM from four distinct analytical replicates. Noteworthy disparities between THP-1 and dTHP-1 are indicated by an asterisk, determined by a Student t-test (***p ≤ 0.001).





**Figure S5. Effect of the AF of human milk and LPS on epithelial, monocytes and macrophages cells viability.** The impact of 0.1% (THP-1) or 0.3% (HT-29 and dTHP-1) AF from raw or pasteurized colostrum and mature milk, both in the absence and presence of 1 µg/mL LPS, on cell viability was evaluated using the MTT method. The data, normalized against the basal conditions (open bars), are presented as the mean ± SEM from six analytical replicates, corresponding to one representative of two independent experiments. The asterisk on the bars indicates significant differences from the control condition, determined by a one-way ANOVA test (***p ≤ 0.001).





**Figure S6. Effect of Holder pasteurization on lactadherin concentration in colostrum.** Lactadherin was quantified via ELISA in the AF of six individual raw (R) and pasteurized (P) samples. The retention percentage was computed using the formula: (CP/CR) x 100, where CP represents the lactadherin concentration in the pasteurized samples and CR signifies the lactadherin concentration in the raw samples. Asterisks denote significant differences (*p≤0.05), determined by the Wilcoxon Matched Pairs Signed Rank test.





**Figure S7. Effect of thermal treatment on sCD14 activity.** The inflammatory response of gut epithelial, macrophage-like, and monocyte-like cells to LPS in the presence of treated (62.5°C, 30 min) or untreated recombinant sCD14 was assessed. The levels of cytokines measured by ELISA are presented as mean ± SEM from three analytical replicates of one representative of two independent experiments. Asterisks on the bars indicate significant differences compared to the control condition (open bars, LPS in the absence of sCD14), determined by the one-way ANOVA test (***p ≤ 0.001). ND = not detected.
